# Very Early Transition to Oral Antibiotics in Uncomplicated Enterobacterales Bloodstream Infections: Effectiveness and Impact on Carbon Footprint Saving

**DOI:** 10.3390/antibiotics14080751

**Published:** 2025-07-25

**Authors:** Aina Mateu, Ana Martínez-Urrea, Clara Gallego, Laura Gisbert, Beatriz Dietl, Mariona Xercavins, Maria López-Sánchez, Silvia Álvarez, Sergi García Rodríguez, Toni Roselló, Josefa Pérez, Esther Calbo, Lucía Boix-Palop

**Affiliations:** 1 Infectious Diseases Department, Hospital Universitari Mútua de Terrassa, 08221 Terrassa, Spain; aina.mateusubira@gmail.com (A.M.); amartinezu@clinic.cat (A.M.-U.); cegallego@mutuaterrassa.cat (C.G.); lgisbert@mutuaterrassa.cat (L.G.); bdgomezluengo@mutuaterrassa.es (B.D.); ecalbo@mutuaterrassa.es (E.C.); 2 Microbiology Department, CatLab, 08232 Viladecavalls, Spain; mxercavins@catlab.cat (M.X.); jperez@catlab.cat (J.P.); 3 Infection Control Nursing Team, Hospital Universitari Mútua de Terrassa, 08221 Terrassa, Spain; mlopezsanchez@mutuaterrassa.cat (M.L.-S.); salvarez@mutuaterrassa.cat (S.Á.); 4 Economic Direction Department, Fundació Assistencial Mútua de Terrassa, 08221 Terrassa, Spain; sergiogarcia@mutuaterrassa.cat; 5 Operations-Quality & Certifications Department, Fundació Assistencial Mútua de Terrassa, 08221 Terrassa, Spain; 6 Infectious Diseases Department, Universitat Internacional de Catalunya, Sant Cugat Campus, 08195 Sant Cugat del Vallès, Spain

**Keywords:** enterobacterales, bloodstream infection, oral stepdown

## Abstract

**Background/Objective**: This study aimed to evaluate the effectiveness of very early oral transition in Enterobacterales bloodstream infections (E-BSIs), identify factors associated with it, compare the effectiveness of different oral options, and assess its economic and ecological benefits. **Methods**: Retrospective, observational cohort study including monomicrobial E-BSI in clinically stable adult patients by day 3 of bacteremia with oral antibiotic options. Transition to oral antibiotics by day 3 or earlier (early oral (EO) group) was compared to later transition or remaining on intravenous therapy (nEO group). Early oral transition-associated factors were analyzed. Oral high-dose beta-lactams (BLs) were compared to quinolones (QLs) or trimethoprim/sulfamethoxazole (TS). Economic and ecological costs were assessed. **Results**: Of 345 E-BSI, 163 (47.2%) were in the EO group, characterized by more urinary tract infections (UTIs) and shorter hospital stays. The nEO group had higher Charlson Comorbidity Index (CCI), extended-spectrum beta-lactamase (ESBL) production, greater source control need, and longer time to clinical stability. There were no significant differences in mortality and relapse. UTIs were associated with early oral transition (OR 2.02, IC 95% 1.18–3.48), while higher CCI (0.85, 0.77–0.95), source control need (0.39, 0.19–0.85), longer time to clinical stability (0.51, 0.39–0.66), and ESBL isolates (0.39, 0.19–0.80) hindered this practice. High-dose BLs and QL/TS were equally effective. Early oral transition resulted in 38.794 KgCO_2_eq reduction and EUR 269,557.99 savings. **Conclusions**: Very early oral transition at day 3 or before in stable E-BSI patients is effective, eco-sustainable, and cost-effective; UTI is related with the early oral switch, while comorbidities, ESBL production, source control need, or longer time to clinical stability hinder this practice.

## 1. Introduction

The management of Enterobacterales bloodstream infections (E-BSIs) has garnered significant interest in recent years due to their increasing prevalence, which is associated with high morbidity and mortality [1]. Traditionally, intravenous (IV) antibiotics for the entire duration of treatment have been the standard of care for treating these infections. This practice is based in studies conducted during a time when few oral options with limited bioavailability were available [2]. However, more recent data, including four randomized controlled trials (RCTs) [3,4,5,6] and numerous observational studies [7,8,9,10,11,12], have demonstrated the effectiveness and safety of transitioning to oral antibiotics after IV treatment. This shift offers advantages, including shorter hospital stays, quicker recovery to baseline condition, improved patient experience, less catheter-related adverse effects [2], reduced healthcare costs, and a potential decrease in the carbon footprint associated with IV treatment and healthcare services.

A recently published RCT [6] demonstrated the non-inferiority of early oral transition in clinically stable E-BSI patients with a controlled source of infection in terms of efficacy and safety. Nevertheless, many questions regarding the transition to oral treatment remain unsolved, including the optimal timing of the oral switch. Previous RCTs [3,4,5] have investigated transitioning to oral antibiotics for E-BSI but involved selective patient groups or delayed the oral switch beyond current recommendations, which suggest a 7-day course for uncomplicated E-BSIs [13,14,15]. More recent trials, testing earlier oral transitions (days 3–5 of IV treatment) [6,9], showed similar efficacy to prolonged IV treatments.

Another critical aspect is the selection of oral antibiotics for the sequential therapy. Traditionally, high-bioavailability antibiotics like quinolones (QLs) or trimethoprim/sulfamethoxazole (TS) were preferred over beta-lactams (BLs) due to concerns about recurrence of bacteremia with BLs [16]. However, subsequent analyses suggest that incorrect dosing of low-bioavailability antibiotics may contribute to these poorer outcomes [17]. Recent studies [6,9,18,19,20] indicate that high-dose BLs are as effective as QL/TS. Additionally, there are safety concerns associated with the use of fluoroquinolones (tendinopathy, cardiac conduction disorders, and Clostridioides difficile infection) and trimethoprim/sulfamethoxazole (renal impairment and hyperkalemia) [21].

Ongoing clinical trials, such as the INVEST trial [22], the GOAT trial (NCT06080698), the SOAB trial (NCT04146922), and the BALANCE + trial (NCT05893147), may provide insights into these aspects of the oral antibiotic treatment for E-BSIs. Meanwhile, we aimed to evaluate the efficacy of a very early transition to oral antibiotics (by day 3 of IV treatment or earlier) in patients with uncomplicated E-BSIs, to identify risk factors associated with this approach and to compare the efficacy of high-dose BL/CEPH vs. QL-TS for the oral treatment of these infections. Additionally, we assessed the reduction in carbon footprint and hospital stay costs associated with this practice.

## 2. Results

### 2.1. Baseline Characteristics and Outcomes

**Figure 1:**
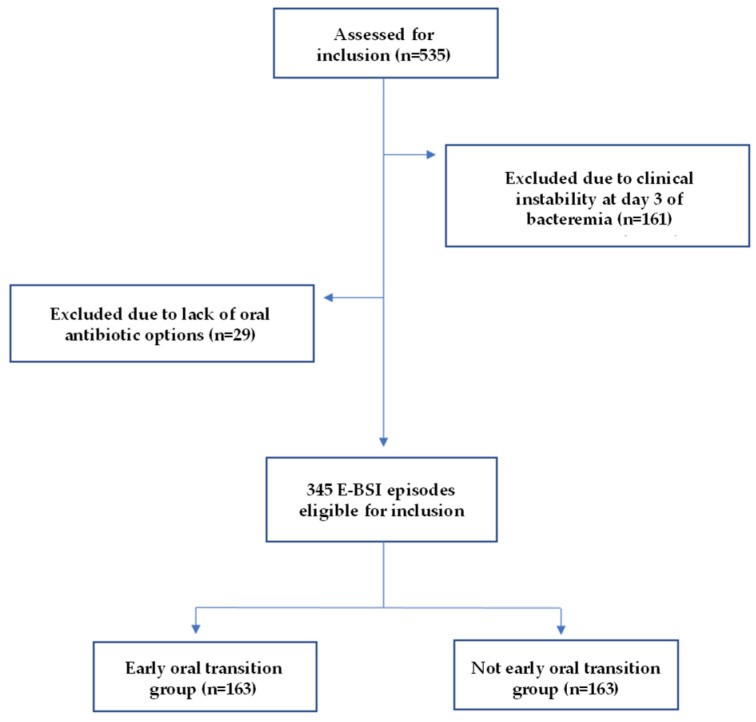
Flowchart of the inclusion process.

A total of 535 E-BSI episodes were reported during the study period. Of these, 345 episodes (64.5% from the initial cohort) fulfilled the inclusion criteria and were included in the analysis: 163 (47.2%) in the early oral group (EO group) and 182 (52.8%) in the non-early oral group (nEO group). Figure 1 shows the flowchart of the inclusion process.

Baseline characteristics and E-BSI details, and the comparison between groups, are presented in Table 1. A total of 31 E-BSI episodes (9%) were diagnosed after the patient was discharged from the Emergency Department, with 28 in the EO group vs. 3 in the nEO group (*p* < 0.001).

The antibiotics used are described in Table S1 in the supplementary material, with no significant differences observed between groups. Despite clinical stability and the availability of oral options, 68 patients continued intravenous treatment, primarily due to clinician decision (70.8%) or clinical situation, mainly ICU stay.

### 2.2. Factors Associated with Very Early Oral Transition

The multivariate logistic regression model used to assess variables associated with very early oral transition is presented in Table 2. Urinary tract infections were the only independent factor significantly associated with early oral transition. Conversely, factors that impeded early oral treatment included a higher Charlson Comorbidity Index (CCI), the need for source control prior to oral transition, longer time to achieve clinical stability, and the presence of extended-spectrum beta-lactamase (ESBL)-producing isolates.

### 2.3. Assessment of the Oral Transition with High Doses of Beta-Lactams and Cephalosporins

Of the 345 E-BSI episodes, 277 (80.3%) were switched to oral antibiotics. Among these, 164 patients (59.2%) received oral QL/TS, 106 patients (38.3%) received BL/CEPH, and 7 patients (2.5%) received fosfomycin. The comparison between patients who received high doses of BL/CEPH and those who received QL/TS is shown in Table S2 in the supplementary material.

The three ESBL cases in the BL/CEPH group were susceptible to amoxicillin/clavulanic acid without other oral options; two presented urinary tract infection (UTI) and one a cellulitis. No differences were found in the intravenous treatment, including its durations and the antibiotics used.

### 2.4. Carbon Footprint Analysis and Hospital Stay Costs

The analysis of the reduction in kilograms of carbon dioxide equivalents (KgCO_2_eq) is presented in Table 3. If an early oral switch had been performed in the nEO-group, the total KgCO_2_eq saved across both groups would have been 82.110 KgCO_2_eq.

Regarding hospital stay costs, a summary of the savings is shown in Table 4. If an early oral transition had been implemented in the nEO group, the total potential savings would have been EUR 567,954.84.

## 3. Discussion

In this study, we found that a very early switch to oral treatment in uncomplicated monomicrobial Enterobacterales bloodstream infections is an effective and safe strategy for selected patients. Factors favoring this practice include having a urinary tract infection, while a higher Charlson index, ESBL-producing isolates, need for source control before the oral transition, and longer time to clinical stability hinder the early oral transition. High doses of beta-lactams or cephalosporins for the oral switch treatment were as effective as quinolones or trimethoprim/sulfamethoxazole in E-BSIs. Additionally, patients in the EO group had shorter hospital stays, leading to reduced healthcare costs and decreased carbon emissions, highlighting its eco-sustainable benefits.

Our study contributes to consolidate the oral route in stable patients with uncomplicated E-BSIs evaluating a very early transition to oral antibiotics within 3 days from the bacteremia onset. Previous studies have shown the efficacy of oral treatment for uncomplicated E-BSIs; however, the duration of IV antibiotics varies widely, between 3 and 7 days. An RCT [5] evaluated the switch to oral antibiotics on day 7 of IV therapy, exceeding current recommendations for treatment duration in uncomplicated E-BSIs [13,14]. Other studies have explored early transitions, with a successful switch to oral antibiotics after 3 days of IV treatment, but with a small sample size [4] or focusing only on quinolones oral therapy [3]. Recently, Omrani et al. [6] conducted an RCT reporting similar clinical failure rates with early transition to oral treatment after 3–5 days of IV antibiotics, and a recent study [10] comparing an early oral switch by day 4 of bacteremia to continued IV treatment found no differences in 90-day mortality between groups.

We explored a very early transition to oral antibiotics, the third day from the bacteremia onset or before. Median IV treatment duration in the EO group was 2 days (interquartile range (IQR) 1–3 days), with 32% receiving one IV dose or none. Both study groups exhibited low rates of in-hospital mortality, 30-day mortality, and bacteremia relapse, without significant differences. It is worth noticing that the CCI in the nEO group differed significantly from patients in the EO group. Taking all this into account suggests the early oral transition’s efficacy and safety in selected clinically stable, uncomplicated E-BSI patients.

Remarkably, in a recent study published by Casado et al. [9], 206 febrile ambulatory patients with low-risk bloodstream infections (BSIs), who received early oral treatment (notably, the majority received no IV antimicrobial doses at all and 27% were switched to oral treatment after a single IV dose), were compared to matched febrile non-bacteremia outpatients. Both cohorts exhibited similar, low rates of 14-day mortality and unplanned re-consultations, suggesting early oral therapy efficacy for bacteremia, even from its onset.

These findings suggest that a very early switch to oral treatment is feasible and no specific duration of IV treatment may be obligatory. Instead, defining criteria for safe and personalized oral transition might be preferable, tailoring the switch timing for each patient [23]. In our cohort of patients, nearly 50% of eligible individuals underwent early oral treatment, contrasting with other studies where such transitions were less frequent, as seen in Omrani’s RCT [6], where only 20.4% of eligible patients (20.4%) were randomized to the oral group.

In our study, the most frequent clinical syndromes and the most frequent Enterobacterales isolates were well-represented in the EO-group, including ESBL producers and multidrug resistance (MDR) isolates, accounting for 9.2% and 15%, respectively, although both mechanisms of resistance were more frequent in the nEO group. Previous observational trials reported lower ESBL or MDR isolates rates in their oral groups (0.2% in the Tingsgard et al. [10] trial, or 2.6% in the Engers et al. [8] trial), making their results less applicable to these microorganisms.

The presence of urinary tract infection was related with early oral transition, consistent with robust literature supporting this practice [4,11,20,24,25]. However, factors that hindered the early oral switch included the need for source control before the oral transition, days to achieve clinical stability, a higher Charlson index score, or the presence of an ESBL-producing isolate. Although these factors are common concerns among physicians considering the oral switch [26], no studies have specifically assessed their impact on oral transition efficacy. Adequate source control and favorable infection progress—being normotensive, afebrile, or having a low Pitt-score—have been described in previous studies as key prerequisites for oral therapy [5,6,10,11]. Another critical consideration is the presence of comorbidities, often indicated by the Charlson index score, which tended to be higher in the IV treatment groups in previous retrospective trials [8,10], reflecting a cautious approach in managing this subgroup, although sub-analysis of elderly patients (≥75 years) [10] showed no significant differences in 90-day mortality among those treated orally. Finally, ESBL-producing isolates are rarely reported. A recent retrospective study found that the presence of MDR-GN bacteria was associated with treatment failure, though the specific resistance mechanism was not described and the treatment failure rate was low (5.7%, 31 patients in total) [12]. Aligned with our results, Engers et al. [8] reported lower rates of ESBL-producing isolates in the oral group (2.6% vs. 10.6%, *p* < 0.001). The higher prevalence of ESBL-producing isolates in the nEO group likely reflects clinical caution in switching to oral therapy in resistant cases.

Regarding the oral antibiotic choice, our study found that high-dose beta-lactams or cephalosporins are equally effective as quinolones or trimethoprim/sulfamethoxazole, challenging the traditional preference for the latter in E-BSI management. The preference for QLs or TS for the treatment of E-BSIs may be due to trials using suboptimal doses of beta-lactams or cephalosporins [17]. Several trials have explored this issue, yielding conflicting results. However, studies using high-dose beta-lactams or cephalosporins reported comparable outcomes to QLs and TS [18,19,20]. Mponponsuo et al. [17] found that while their general analysis favored highly bioavailable antibiotics, subgroup analysis comparing standard doses of highly bioavailable antibiotics with high doses of less bioavailable antibiotics showed comparable primary outcomes—a composite of mortality, recurrent BSIs with the same pathogen (genus and species), and all-cause readmission at 90 days post-discharge—with odds ratio of 0.97 (95% CI, 0.59–1.60). Other studies also showed successful outcomes with high-dose beta-lactams [6,9,12]. Based on this previous data and our findings, beta-lactams and cephalosporins may be viable alternatives to QLs and TS for stability, though rigorous trials are warranted to further investigate this matter.

Our study uniquely quantified the environmental impact of early transition to oral treatment, estimating a 38.75 KgCO_2_eq reduction during the study period. If all eligible patients had transitioned by day 3, this reduction could have been 82.01 KgCO_2_eq, equivalent to a round-trip high-speed train journey from Barcelona to Madrid. Given carbon dioxide’s role in the greenhouse effect and climate change, strategies reducing healthcare emissions are increasingly relevant and warrant further investigation.

The treatment failure rate in our study was 2.9% overall and 1.9% in the EO group, lower than those previously reported by other authors [12,25], probably due to the exclusion of unstable patients on day 3, a high proportion of urinary tract infections as the E-BSI source, and *E. coli* isolates representing less severe patients.

Additionally, in our cohort of patients, hospital stay was remarkably shorter in the EO group (3 vs. 8 days, *p* < 0.001), paralleling findings from previous observational trials (5 vs. 7 days in the Tamma et al. [11] trial, 4 vs. 12 days in the Engers et al. [8] trial, and 6 vs. 9 days in the Omrani et al. [6] trial). Cost analyses indicated substantial savings with early oral transition due to reduced hospitalization and antibiotic costs. The early transition to oral treatment represented a savings of EUR 269,557.99 during the study, and the total potential savings (EO group plus nEO group) would have been EUR 567,954.84. These results must be nonetheless interpreted carefully, as patients differed significantly in some relevant aspects between groups, like the higher CCI in the nEO group, which may have contributed to the longer hospital stay, and therefore the potential savings calculated among this group would be lower. Previous trials analyzing the cost-effectiveness of this strategy regarding other infectious syndromes have been performed, with similar results [27,28]. However, to our knowledge very few studies have been published regarding the oral treatment of E-BSIs, mainly comprising small trials aligning with our findings [3,29].

The major strength of this study is that it includes a well-defined cohort of patients with representation across various clinical syndromes and isolates, including ESBL-producing and MDR strains. Moreover, we defined a very early oral transition more ambitiously than previous studies and achieved a high transition to oral antibiotics rate. Finally, we assessed some of the most relevant actual issues related to the oral treatment of E-BSIs, like the selection of the oral regimen, the evaluation of hospital costs, and the impact on the carbon footprint.

The main limitations of the present study are (a) the retrospective and observational design, requiring cautious interpretation of the results despite having excluded clinically unstable patients and patients with no oral options; (b) the study was limited to infections caused by Enterobacterales; therefore, we cannot extrapolate our results to other Gram-negative microorganisms; in the same line, high-inoculum infections like central nervous system infections are very poorly represented; (c) there were imbalances between both groups of some characteristics that may affect the outcomes; (d) high doses of beta-lactams and cephalosporins were standard doses, and were not calculated according to height and weight as this data was not included in the data-gathering process; (e) given the retrospective nature of our study and the diversity of managing teams, we were unable to ascertain whether the availability of active oral agents influenced the decision to transition to oral therapy. Finally, our study was conducted in a specific geographical area and the results cannot be extrapolated to other settings.

## 4. Materials and Methods

### 4.1. Study Design, Setting, and Patients

A retrospective observational cohort study was conducted at Hospital Universitari Mútua de Terrassa, a 400-bed teaching hospital in Barcelona, Spain. Since 1989, all consecutive episodes of clinically significant BSI in adults (≥16 years old) have been prospectively recorded by the Bloodstream Infection Surveillance Program, a multidisciplinary team that performs prospective clinical follow-ups on all patients with documented bacteremia.

The current study included all consecutive episodes of monomicrobial E-BSI that were susceptible to at least one antibiotic with available oral formulation in patients with clinical stability on day 3 of the bacteremia, between January 2021 and December 2022. Patients could be included multiple times if they experienced E-BSI at different times. We compared patients who were switched to oral antibiotics by day 3 of bacteremia or earlier (EO group) with those who had a later oral transition or received full IV treatment (nEO group ). Patients were followed-up for 30 days.

Informed consent was waived since this was a retrospective observational study without direct patient interaction. The study followed STROBE recommendations to strengthen the reporting of the results (Supplementary Table S3) [30].

### 4.2. Variables and Definitions

Clinical stability was defined as having a heart rate inferior than 100 beats per minute, a respiratory rate inferior than 24 breaths per minute, a body temperature inferior than 37.2 degrees Celsius, a systolic blood pressure above 90 mmHg, an oxygen saturation above 90%, a proper consciousness level, and adequate oral tolerance, as defined by Halm et al. [31]. Efficacy was measured by all-cause in-hospital mortality and all-cause 30-day mortality. Recurrence was defined as readmission due to an infection caused by the same microorganism within 30 days after the initial BSI.

The choice of oral antibiotics was at the clinician’s discretion, following local recommendations, that recommend high doses of oral BL/CEPH—specifically, amoxicillin 1000 mg/8 h, amoxicillin/clavulanic 875/125 mg/8 h, and cefuroxime 500 mg/8 h, according to a recently published consensus [32].

Additional clinical variables collected included age, gender, comorbidities based on the CCI [33], immunosuppression status, infection acquisition site based on Friedman et al. definitions [34], the source of BSI according to CDC criteria [35], severity of the BSI according to the Pitt-score [36], sepsis and shock according to standard definitions [37], analytical data on the day of bacteremia, the day of bacteremia, the day the clinician was informed by the Microbiology Department, etiology of the BSI, antibiotic treatment and its duration, appropriateness of empiric antibiotic treatment—defined as receiving an antibiotic to which the isolate was susceptible—requirement for infection source control, and length of hospital stay. Data were collected from medical charts and electronic medical records.

### 4.3. Microbiology

The recommendations of the Spanish Society of Infectious Diseases and Clinical Microbiology (SEIMC) were followed for performing, processing, and interpreting blood cultures [38]. Antibiotic susceptibility and ESBL production were interpreted according to the European Committee on Antimicrobial Susceptibility Testing (EUCAST), and the presence of MDR was interpreted according to Magiorakos et al. definitions [39].

### 4.4. Carbon Footprint and Economic Costs

The carbon footprint was measured in KgCO_2_eq. The total carbon footprint generated by each group of patients (EO and nEO) was calculated by considering, for each antibiotic per patient, the type and dose of the antibiotic, the KgCO_2_eq generated from its production (extrapolated from a study by Weisz et al. in Australia in 2020 [40]), and the KgCO_2_eq generated from the production of each intravenous kit (0.0048 KgCO_2_eq per kit, data obtained from an internal study based on PAS 2050 [41] and ISO 14067 [42] detailed in supplementary material). Data on the carbon footprint from the hospital stay was unavailable, so it was not included in the calculation.

Healthcare costs were measured in euros (EUR), encompassing the costs of hospitalization and IV treatment. Details on the methodology used to assess the carbon footprint and the economic costs are provided in supplementary material.

### 4.5. Statistical Analysis

All statistical analyses were conducted using STATA RELEASE 18 software (StataCorp LP, College Station, TX, USA). Categorical variables are presented as counts and percentages, while continuous variables are presented as medians and IQR. Univariate analysis was performed using the Chi-square test or Fisher’s exact test in categorical variables, and the Student’s *t*-test or Mann–Whitney U-test for continuous variables, as convenient. Missing data are reported in the tables. A two-sided *p* value of <0.05 was considered statistically significant.

Multivariate analysis was performed using logistic regression. Starting with all variables that showed a trend towards an association (*p* < 0.2), a best subset regression procedure was used to identify the most suitable and parsimonious multivariate model, defined as the one with the lowest Akaike Information Criterion (AIC), a widely recognized measure of model fit [43]. Differences were considered statistically significant at a two-sided *p* value of <0.05 level.

## 5. Conclusions

In conclusion, very early oral antibiotic transition in selected patients with uncomplicated Enterobacterales bloodstream infections is an effective and safe strategy, while demonstrating eco-sustainability and cost-effectiveness. Furthermore, the use of high doses of BL/CEPH is as effective as QL/TS in the oral switching for E-BSI treatment.

## Figures and Tables

**Table 1:** Baseline characteristics and outcomes of Enterobacterales bloodstream infections, and comparison between early oral transition group vs. non-early oral transition group.

	EO Group (*n* = 163) (%)	nEO Group (*n* = 182) (%)	*p*
Sex (male)	85 (52.2)	93 (51.1)	0.85
Age ^a^	75 (64–84)	77 (67–86)	0.06
Charlson index ^a^ (*n* = 344)	4 (3–6)	5 (4–6)	0.002
Diabetes Mellitus	33 (20.3)	49 (26.9)	0.146
Immunosuppression ^b^	14 (8.6)	22 (12.1)	0.29
Corticosteroids	6 (42.9)	6 (27.3)	0.33
Biologic treatment	3 (21.4)	6 (27.3)	1.00
Neutropenia	1 (7.1)	1 (4.6)	1.00
Solid organ transplant	2 (14.3)	1 (4.6)	0.55
Hematopoietic cell transplant	0 (0.0)	3 (13.6)	0.27
Chemotherapy	5 (35.7)	10 (45.5)	0.73
AIDS	0 (0.0)	0 (0.0)	NA
Bacteremia characteristics			
Classification according to acquisition-site (*n* = 339)			
Community-acquired	99 (61.5)	97 (54.5)	0.19
Health care-associated infection	45 (28.0)	51 (28.7)	0.89
Nosocomial	17 (10.6)	30 (16.9)	0.09
Source of infection			
Urinary-tract	129 (79.1)	118 (64.8)	0.003
Biliary-tract	25 (15.3)	39 (21.4)	0.15
Unknown	8 (4.9)	8 (4.4)	0.82
Others ^c^	1 (0.6)	17 (9.3)	<0.001
Initial Pitt-score ^a^ (*n* = 333)	0 (0–0)	0 (0–1)	0.14
Sepsis (*n* = 341)	29 (17.9)	43 (24.0)	0.17
Septic shock (*n* = 341)	3 (1.9)	11 (6.2)	0.06
ICU admission (*n* = 339)	4 (2.5)	10 (5.7)	0.18
Initial temperature ^a^ (*n* = 333)	37.8 (37.2–38.5)	37.8 (37·2–38.3)	0·51
Initial Leukocyte count ^a^(*n* = 343)	11.9 (8.9–16.3)	11.5 (8.3–15.0)	0.38
Initial C-RP ^a^ (*n* = 337)	86.3 (37.0–175.0)	98.3 (38.2–174.9)	0.53
Initial PCT ^a^ (*n* = 259)	0.9 (0.4–3.3)	1.6 (0.6–6.7)	0.04
Microbiology			
Days from blood culture extraction to clinician information ^a^ (*n* = 341)	1 (1–2)	1 (1–2)	0.46
Microorganism			
*E. coli*	121 (74.2)	141 (77.5)	0.53
*K.pneumoniae*	28 (17.2)	32 (17.6)	0.92
Others ^d^	14 (8.6)	9 (5.0)	0.18
Any resistance mechanism	16 (9.8)	35 (19.2)	0.01
ESBL	15 (9.2)	33 (18.1)	0.02
Others ^e^	1 (0.6)	2 (1.1)	1.00
Multidrug resistance	25 (15.3)	31 (17.0)	0.67
Antibiotic treatment			
Any empiric antibiotic	158 (96.9)	180 (98.9)	0.26
Appropriate empiric antibiotic (*n* = 328)	139 (91.5)	152 (86.4)	0.15
Days from bacteremia to first antibiotic administration ^a^	0 (0–1)	0 (0–0)	0.20
Days of intravenous antibiotics ^a^ (*n* = 344)	2 (1–3)	6 (4–8)	<0.001
Total length of antibiotic treatment ^a^ (*n* = 344)	10 (7–14)	10 (7–13)	0.56
Source control before oral treatment (*n* = 339)			
Yes	12 (7.5)	38 (21.4)	<0.001
No	4 (2.5)	5 (2.8)	1.00
Not indicated	142 (88.2)	128 (71.9)	<0.001
Outcomes			
Days to clinical stability from antibiotic initiation ^a^	1 (1–2)	2 (1–3)	<0.001
Phlebitis during admission (*n* = 334)	3 (1.9)	10 (5.8)	0.09
Length of hospital stay ^a^ (*n* = 333)	3 (1–5)	8 (5–14)	<0.001
In-hospital mortality	2 (1.2)	7 (3.9)	0.18
30-day mortality	2 (1.2)	5 (2.8)	0.45
Re-admission due to a new infection (*n* = 327)	10 (6.3)	26 (15.4)	0.009
Re-admission due to relapse of the E-BSI (*n* = 322)	3 (1.9)	7 (4.2)	0.34

^a^ Median (interquartile range). ^b^ Patients could have more than one mechanism of immunosuppression at a time. ^c^ Other sources of infection: intra-abdominal, catheter-related, pneumonia, skin and soft tissue, endovascular, central nervous system, febrile neutropenia. ^d^ Other microorganisms: *Citrobacter* spp., *Enterobacter* spp., *Klebsiella oxytoca*, *Proteus mirabillis*, *Serratia marcescens*. ^e^ Other resistance mechanisms: 1 AmpC (EO group)/1 AmpC, 1 Oxa-48 (nEO group).

**Table 2:** Multivariate analysis—factors associated with an early oral transition.

Variable	Odds Ratio	95% Confidence Interval	*p* Value
Charlson index	0.85	0.77–0.95	0.003
Urinary tract infection	2.02	1.18–3.48	0.01
ESBL-producing isolate	0.39	0.19–0.80	0.01
Source control previous to the oral transition	0.39	0.19–0.85	0.02
Days to clinical stability	0.51	0.39–0.66	<0.001

**Table 3:** Analysis of carbon footprint.

	Carbon Footprint		
	Total KgCO_2_eq Per Group	Mean KgCO_2_eq Per Patient	Absolute Savings (KgCO_2_eq) ^a^
EO group (*n* = 163)	44.279	0.272	−38.794
nEO group (*n* = 182)	92.704	0.509	−43.316
Difference		−0.238	

^a^ Absolute savings (carbon footprint) = −0·238 × number of patients (each group). The EO group represents the real savings, and the nEO group represents the potential savings obtained if the early oral transition had been performed in this group.

**Table 4:** Analysis of hospital costs and savings.

	Hospital Stay Cost				Antibiotics Cost				Intravenous Kit Cost				Total Savings (EUR)
	Median Hospital Stay (Days)	Cost Per Day of Hospital Stay (€)	Mean Total Cost Per Patient (EUR)	Absolut Savings (EUR) ^a^	Total Days of Antibiotics Per Group	Total Cost of Antibiotics Per Group (EUR)	Mean Cost of Antibiotics Per Day Per Patient (EUR)	Absolute Savings (EUR) ^b^	Total Days of Intravenous Kit Use Per Group	Total Cost of Intravenous Kit Per Group (EUR)	Mean Cost of Intravenous Kit Per Day Per Patient (EUR)	Absolute Savings (EUR) ^c^	
EO group (*n* = 163)	4	266.22	1064.88	−260,363.32	1899	5189.28	2.73	−8736.46	319	1226.92	3.85	−458.21	−269,557.99
nEO group (*n* = 182)	10	266.22	2662.2	−290,712.42	1473	10,801.80	7.33	−6776.63	632	3338.56	5.28	−907.80	−298,396.85
Difference			−1597.32				−4.60				−1.44		

^a^ Absolute savings (hospital stay cost) = EUR −1597.32 × number of patients (each group). The EO group represents the real savings, and the nEO group represents the potential savings obtained if the early oral transition had been performed in this group. ^b^ Absolute savings (antibiotic cost) = EUR −4.60 × total days of antibiotic (each group). The EO group represents the real savings, and the nEO group represents the potential savings obtained if the early oral transition had been performed in this group. ^c^ Absolute savings (intravenous kit cost) = EUR −1.44 × total days of intravenous kit use (each group). The EO group represents the real savings, and the nEO group represents the potential savings obtained if the early oral transition had been performed in this group.

## Data Availability

Upon request, individual deidentified participant data and the data dictionary will be provided upon publication. Only scientifically sound requests for data will be considered. Researchers are requested to contact with the corresponding author in order to submit the petitions.

## Supplementary Materials

The following supporting information can be downloaded at: https://www.mdpi.com/article/10.3390/antibiotics14080751/s1, Table S1. Summary of antibiotic treatment for Enterobacterales bloodstream infections; Table S2. Baseline characteristics and outcomes of patients treated with oral antibiotics, and comparison between high doses of beta-lactams/cephalosporins vs. quinolones or trimethoprim/sulfamethoxazole; Table S3. STROBE statement—checklist of items that should be included in reports of observational studies.

## Abbreviations

The following abbreviations are used in this manuscript:

AIC	Akaike Information Criterion
BLs	Beta-lactams
BSIs	Bloodstream infections
CCI	Charlson Comorbidity Index
E-BSIs	Enterobacterales bloodstream infections
EO group	Early oral group
ESBL	Extended-spectrum beta-lactamase
ESCMID	European Society of Clinical Microbiology and Infectious Diseases
EUCAST	European Committee on Antimicrobial Susceptibility Testing
IQR	Interquartile range
IV	Intravenous
KgCO_2_eq	Kilograms of carbon dioxide equivalents
MDR	Multidrug resistance
nEO group	Non-early oral group
QLs	Quinolones
RCTs	Randomized controlled trials
SEIMC	Spanish Society of Infectious Diseases and Clinical Microbiology
TS	Trimethoprim/sulfamethoxazole
UTI	Urinary tract infection
