# Supplementary material for: Very Early Transition to Oral Antibiotics in Uncomplicated Enterobacterales Bloodstream Infections: Effectiveness and Impact on Carbon Footprint Saving
Source: Antibiotics (Basel). 2025 Jul 25;14(8):751. doi: 10.3390/antibiotics14080751 (PMC12382972; doi:10.3390/antibiotics14080751)
Supplement: Supplementary file 1 [file antibiotics-14-00751-s001.zip › antibiotics-3748311-supplementary.docx]

**SUPPLEMENTARY MATERIAL**

1. **Description of the methodology used to assess the carbon footprint and the economic costs**

The KgCO_2_eq generated from the production of each intravenous kit was 0·0048 KgCO_2_eq per kit; data obtained from an internal study based on PAS 2050 (1) and ISO 14067 (2).

By knowing the total amount of KgCO_2_eq per group and the total number of patients in each group, we computed the median KgCO_2_eq generated per patient per group; the difference between groups represented the ”saving of carbon footprint per patient of the EO group”. We therefore calculated the real reduction of carbon footprint generated in the EO group by multiplying the median saving of carbon footprint per patient in the EO group per the number of patients in this group. We also assessed the potential reduction that would have been obtained if this practice had been applied to the nEO group.

Regarding the economic costs, we again calculated the real savings generated by the early oral switch and the potential savings of the nEO group. The total costs per group included the costs of the hospital stay and the cost of the consumed antibiotics (the cost of the antibiotic itself along with the cost of the IV kit). We used a similar method as the explained above; we took into account the median cost per day of hospital stay at our hospital (EUR 266·22 per day, internal studies) and the median hospital stay per group in order to calculate the median cost of hospital stay per patient of each group; the difference between the two groups represented the “median saving attributed to each patient of the EO group”. By multiplying this value per the total number of patients in the EO group, we obtained the real economic savings in the EO group, and by multiplying it per the total number of patients in the nEO group, we obtained the potential benefit in this group. The total days of antibiotics are the sum of the total antibiotics that each of the patients in each of the groups (EO group and nEO group) had received. The costs derived from the antibiotic and the iv kits consumption were computed with the same method from knowing the total days of antibiotics/total number of IV kits and the total cost of antibiotics/IV kits per group.

1. British Standards Institution. PAS 2050: Specification for the assessment of the life cycle greenhouse gas emissions of goods and services. 2nd ed. London: BSI; 2011.
2. International Organization for Standardization. ISO 14067: Greenhouse gases — Carbon footprint of products — Requirements and guidelines for quantification. Geneva: ISO; 2018.

Table S1. Summary of antibiotic treatment for Enterobacterales bloodstream infections.

|  | EO group (n=158) | nEO group (n=180) | p |
| --- | --- | --- | --- |
| Empiric intravenous antibiotics  Ceftriaxone  Amoxicillin/clavulanic acid  Piperacillin/tazobactam  Amikacin  Meropenem  Ertapenem  Ciprofloxacin  Imipenem  Metronidazole  Aztreonam  Azithromycin  Ceftriaxone/metronidazole  Cefotaxime/metronidazole  Amoxicillin/clavulanic acid/amikacin  Piperacillin/tazobactam/amikacin  Ceftriaxone/amikacin  Meropenem/vancomycin  Meropenem/linezolid  Others | 79 (50·0)  5 (3·2)  4 (2·5)  1 (0·6)  2 (1·3)  11 (7·0)  2 (1·3)  5 (3·2)  2 (1·3)  1 (0·6)  1 (0·6)  1 (0·6)  3 (1·9)  21 (13·3)  1 (0·6)  12 (7·6)  0 (0·0)  1 (0·6)  6 (4·0) | 53 (29·4)  10 (5·6)  11 (6·1)  0 (0·0)  9 (5·0)  10 (5·6)  1 (0·6)  8 (4·4)  0 (0·0)  0 (0·0)  0 (0·0)  13 (7·2)  13 (7·2)  22 (12·2)  5 (2·8)  9 (5·0)  3 (1·7)  4 (2·2)  9 (5·1) | <0·001  0·43  0·12  0·47  0·07  0·59  0·60  0·58  0·22  0·47  0·47  0·002  0·02  0·77  0·22  0·32  0·25  0·38  0·62 |
|  | EO group (n=162) | nEO group (n=114) |  |
| Oral antibiotics  Amoxicillin/clavulanic  Amoxicillin  Cefuroxime  Ciprofloxacin  Cotrimoxazole  Fosfomycin  Ciprofloxacin/metronidazole | 10 (6·2)  6 (3·7)  39 (23·9)  82 (50·6)  24 (14·8)  1 (0·6)  1 (0·6) | 16 (14·0)  4 (3·5)  31 (27·2)  49 (43·0)  8 (7·0)  6 (5·3)  0 (0·0) | 0·03  1·00  0·54  0·21  0·05  0·02  1·00 |

Table S2. Baseline characteristics and outcomes of patients treated with oral antibiotics, and comparison between high doses of beta-lactams/cephalosporins vs. quinolones or trimethoprim/sulfamethoxazole.

|  | BL-CEPH (n=106) | QL-T/S (n=164) | p |
| --- | --- | --- | --- |
| Sex (male) | 49 (46·2) | 89 (54·3) | 0·20 |
| Age^a^ | 77 (65 – 85) | 75 (64 – 84) | 0·38 |
| Charlson index^a^ | 5 (3 – 6) | 4 (3 – 6) | 0·39 |
| Immunosuppression | 9 (8·5) | 16 (9·8) | 0·73 |
| Bacteremia characteristics |  |  |  |
| Classification according to acquisition-site (n=266)  Community-acquired  Health care-associated infection  Nosocomial | 67 (63·2)  25 (23·6)  12 (11·3) | 99 (60·4)  46 (28·1)  17 (10·4) | 0·64  0·42  0·81 |
| Infection origin  Urinary-tract  Biliary-tract  Unknown  Others | 71 (67·0)  26 (24·5)  6 (5·7)  3 (2·8) | 134 (81·7)  23 (14·0)  4 (2·4)  3 (1·8) | 0·006  0·03  0·20  0·68 |
| Initial Pitt-score^a^ | 0 (0 – 0) | 0 (0 – 1) | 0·06 |
| Sepsis (n=268) | 17 (16·0) | 37 (22·8) | 0·18 |
| Septic shock (n=267) | 5 (4·7) | 6 (3·7) | 0·76 |
| ICU admission (n=265) | 3 (2·9) | 6 (3·7) | 1·00 |
| Initial temperature^a^ | 37·8 (37 – 38·4) | 37·9 (37·3 – 38·5) | 0·24 |
| Initial Leukocyte count^a^ | 11·1 (8·4 – 15·6) | 12·0 (9·0 – 16·6) | 0·26 |
| Initial C-RP^a^ | 101·6 (28·0 – 172·7) | 86·3 (38·3 – 175·0) | 0·86 |
| Initial PCT^a^ | 1·2 (0·4 – 4·4) | 0·9 (0·4 – 4·3) | 0·67 |
| Microbiology |  |  |  |
| Days from blood culture extraction to clinician information^a^ | 1 (1 – 2) | 1 (1 – 2) | 0·77 |
| Microorganism  *E.coli*  *K.pneumoniae*  Others | 84 (79·3)  15 (14·2)  7 (6·6) | 124 (75·6)  27 (16·5)  13 (7·9) | 0·49  0·61  0·69 |
| Resistance mechanism  ESBL  Other^b^ | 3 (2·8)  0 (0·0) | 20 (12·2)  2 (1·2) | 0·007  0·52 |
| Multidrug resistance | 12 (11·3) | 27 (16·5) | 0·24 |
| Antibiotic treatment |  |  |  |
| Any empiric antibiotic | 105 (99·1) | 164 (100·0) | 0·39 |
| Appropriate empiric antibiotic (n=254) | 93 (95·9) | 141 (89·8) | 0·10 |
| Days from bacteremia to first antibiotic administration^a^ | 0 (0 – 0) | 0 (0 – 0) | 0·69 |
| Days of intravenous antibiotics^a^ | 3 (1 – 5) | 3 (2 – 4) | 0·81 |
| Total antibiotic treatment^a^ | 10 (7 – 12) | 11 (8 – 16) | 0·03 |
| Source control before oral treatment (n=262)  Yes  No  Not indicated | 12 (11·3)  2 (1·9)  89 (84·0) | 23 (14·0)  6 (3·7)  130 (79·3) | 0·52  0·49  0·34 |
| Outcomes |  |  |  |
| Phlebitis during admission (n=263) | 3 (2·9) | 6 (3·7) | 1·00 |
| Length of hospital stay^a^ | 4 (1 – 7) | 5 (3 – 8) | 0·07 |
| In-hospital mortality | 1 (0·9) | 2 (1·2) | 1·00 |
| 30-day mortality | 3 (2·8) | 2 (1·2) | 0·38 |
| Re-admission due to a new infection (n=260) | 8 (8·0) | 14 (8·8) | 0·83 |
| Re-admission due to relapse of the E-BSI (n=253) | 2 (2·0) | 2 (1·3) | 1·00 |
| ^a^ Median (interquartile range)  ^b^ Other resistance mechanisms: 2 AmpC | | | |

Table S3. STROBE statement—checklist of items that should be included in reports of observational studies.

|  | Item No. | Recommendation | Page  No. | Relevant text from manuscript |
| --- | --- | --- | --- | --- |
| **Title and abstract** | 1 | (*a*) Indicate the study’s design with a commonly used term in the title or the abstract | 1 |  |
|  |  | (*b*) Provide in the abstract an informative and balanced summary of what was done and what was found | 1 |  |
| Introduction | | | |  |
| Background/rationale | 2 | Explain the scientific background and rationale for the investigation being reported | 2 |  |
| Objectives | 3 | State specific objectives, including any prespecified hypotheses | 2 |  |
| Methods | | | |  |
| Study design | 4 | Present key elements of study design early in the paper | 11 |  |
| Setting | 5 | Describe the setting, locations, and relevant dates, including periods of recruitment, exposure, follow-up, and data collection | 11 |  |
| Participants | 6 | (*a*) *Cohort study*—Give the eligibility criteria, and the sources and methods of selection of participants. Describe methods of follow-up  *Case-control study*—Give the eligibility criteria, and the sources and methods of case ascertainment and control selection. Give the rationale for the choice of cases and controls  *Cross-sectional study*—Give the eligibility criteria, and the sources and methods of selection of participants | 12 |  |
|  |  | (*b*) *Cohort study*—For matched studies, give matching criteria and number of exposed and unexposed  *Case-control study*—For matched studies, give matching criteria and the number of controls per case | N/A |  |
| Variables | 7 | Clearly define all outcomes, exposures, predictors, potential confounders, and effect modifiers. Give diagnostic criteria, if applicable | 12 |  |
| Data sources/ measurement | 8* | For each variable of interest, give sources of data and details of methods of assessment (measurement). Describe comparability of assessment methods if there is more than one group | 12 |  |
| Bias | 9 | Describe any efforts to address potential sources of bias | N/A |  |
| Study size | 10 | Explain how the study size was arrived at | N/A |  |

| Quantitative variables | 11 | Explain how quantitative variables were handled in the analyses. If applicable, describe which groupings were chosen and why | 12,13 |  |
| --- | --- | --- | --- | --- |
| Statistical methods | 12 | (*a*) Describe all statistical methods, including those used to control for confounding | 12,13 |  |
|  |  | (*b*) Describe any methods used to examine subgroups and interactions | 12,13 |  |
|  |  | (*c*) Explain how missing data were addressed | 12,13 |  |
|  |  | (*d*) *Cohort study*—If applicable, explain how loss to follow-up was addressed  *Case-control study*—If applicable, explain how matching of cases and controls was addressed  *Cross-sectional study*—If applicable, describe analytical methods taking account of sampling strategy | N/A |  |
|  |  | (*e*) Describe any sensitivity analyses | N/A |  |
| Results | | | | |
| Participants | 13* | (a) Report numbers of individuals at each stage of study—e.g. numbers potentially eligible, examined for eligibility, confirmed eligible, included in the study, completing follow-up, and analyzed | 3 |  |
|  |  | (b) Give reasons for non-participation at each stage | 3 |  |
|  |  | (c) Consider use of a flow diagram | 3 |  |
| Descriptive data | 14* | (a) Give characteristics of study participants (e.g. demographic, clinical, social) and information on exposures and potential confounders | 3-5 |  |
|  |  | (b) Indicate number of participants with missing data for each variable of interest | 3-5 |  |
|  |  | (c) *Cohort study*—Summarize follow-up time (e.g., average and total amount) | 3-5 |  |
| Outcome data | 15* | *Cohort study*—Report numbers of outcome events or summary measures over time | 3-5 |  |
|  |  | *Case-control study—*Report numbers in each exposure category, or summary measures of exposure | N/A |  |
|  |  | *Cross-sectional study—*Report numbers of outcome events or summary measures | N/A |  |
| Main results | 16 | (*a*) Give unadjusted estimates and, if applicable, confounder-adjusted estimates and their precision (e.g., 95% confidence interval). Make clear which confounders were adjusted for and why they were included | 3-5 |  |
|  |  | (*b*) Report category boundaries when continuous variables were categorized | N/A |  |
|  |  | (*c*) If relevant, consider translating estimates of relative risk into absolute risk for a meaningful time period | N/A |  |

| Other analyses | 17 | Report other analyses done—e.g. analyses of subgroups and interactions, and sensitivity analyses | N/A |  |
| --- | --- | --- | --- | --- |
| Discussion | | | | |
| Key results | 18 | Summarize key results with reference to study objectives | 9 |  |
| Limitations | 19 | Discuss limitations of the study, taking into account sources of potential bias or imprecision Discuss both direction and magnitude of any potential bias | 11 |  |
| Interpretation | 20 | Give a cautious overall interpretation of results considering objectives, limitations, multiplicity of analyses, results from similar studies, and other relevant evidence | 9-11 |  |
| Generalizability | 21 | Discuss the generalizability (external validity) of the study results | 11 |  |
| Other information | |  | | |
| Funding | 22 | Give the source of funding and the role of the funders for the present study and, if applicable, for the original study on which the present article is based | 13 |  |

*Give information separately for cases and controls in case-control studies and, if applicable, for exposed and unexposed groups in cohort and cross-sectional studies.

**Note:** An Explanation and Elaboration article discusses each checklist item and gives methodological background and published examples of transparent reporting. The STROBE checklist is best used in conjunction with this article (freely available on the Web sites of PLoS Medicine at http://www.plosmedicine.org/, Annals of Internal Medicine at http://www.annals.org/, and Epidemiology at http://www.epidem.com/). Information on the STROBE Initiative is available at www.strobe-statement.org.
